# Draft Genome Sequence of *Nitrincola* sp. Strain A-D6, an Arsenic-Resistant Gammaproteobacterium Isolated from a Salt Flat

**DOI:** 10.1128/genomeA.01144-14

**Published:** 2014-11-20

**Authors:** Natalia Valdés, Javier Rivera-Araya, Jonathan Bijman, Lorena Escudero, Cecilia Demergasso, Sebastián Fernández, Alonso Ferrer, Renato Chávez, Gloria Levicán

**Affiliations:** a Departamento de Biología, Facultad de Química y Biología, Universidad de Santiago, Santiago, Chile; b Centro de Biotecnología, Universidad Católica del Norte, Antofagasta, Chile; c Centro de Investigación Científico y Tecnológico para la Minería (CICITEM), Antofagasta, Chile

## Abstract

We report *Nitrincola* sp. strain A-D6, which was characterized as an arsenic-resistant bacterium isolated from the Ascotán Salt Flat in northern Chile. The size of the genome is 3,795,776 bp, with a G+C content of 49.96%. Genes for the arsenic-resistant Ars system and arsenic oxidation have been encoded.

## GENOME ANNOUNCEMENT

Arsenic (As) is a ubiquitous element present in the environment in different forms (1). Inorganic arsenic, most often in trivalent or pentavalent form, is the most abundant species of As in nature and is commonly present in soil, water, and food (2). This metalloid is extremely toxic to living organisms and its toxicity is primarily based on its chemical speciation. However, some microorganisms cope with arsenic toxicity in a variety of different ways (1, 3, 4). Such microorganisms therefore play an important role in the arsenic geocycle (5).

*Nitrincola* sp. strain A-D6 was isolated from Salar de Ascotán, a circum-neutral and arsenic-containing salt flat environment in northern Chile. The *Nitrincola* genus comprises aerobic and Gram-negative bacteria belonging to the *Gammaproteobacteria* subdivision (6). This report can shed light on the molecular mechanisms involved in arsenic metabolism in this bacterium and its role in the biogeochemical cycle of arsenic in this extreme environment.

The genome of strain A-D6 was sequenced using the Ion Torrent PGM platform and single-end libraries. The low-quality sequences were examined by FastQC (version 0.10.1; Babraham Institute [http://www.bioinformatics.bbsrc.ac.uk/projects/fastqc/]) and then trimmed by Trimmomatic version 0.32 (7) before assembly. The trimmed sequence were assembled *de novo* using a coverage assembled of 40×, with Mira assembler version 4.0.2 (8) and the CAP3 (9) program. This resulted in 117 contigs. Contig sizes range from 850 to 121,978 bp (*N*_50_, 52,683 bp). The length of the total draft genome of *Nitrincola* sp. strain A-D6 is 3,795,776 bp, with a G+C content of 49.96%.

The annotation of the assembled genome was submitted to the RAST (Rapid Annotation using Subsystem Technology) server (10). The tRNA genes were detected by tRNAscan-SE version 1.23 (11) and the rRNA with RNAmmer (12). The draft genome was shown to encode 43 tRNA sequences, one complete 5S-16S-23S operon, and 4,401 protein-coding genes, 22.27% of which were assigned hypothetical functions. This hypothetical set was assigned to 4 clusters of orthologous groups that comprise a metabolic cluster with 662 genes, a genetic information processing cluster with 187 genes, an environmental information processing cluster with 272 genes, and a cellular process cluster with 99 genes.

The genes responsible for arsenic metabolism include arsenite oxidase small subunit (*aioA*) and large subunit (*aioB*), and an *azu* gene encoding for the physiological electron acceptor azurin (13). The genes responsible for arsenic resistance include an Ars system (*arsRDACH*). ArsC from the Ars system is a cytoplasmic arsenate reductase that reduces As(V) to As(III) (1). ArsD represses upper expression levels of the *ars* operon in the absence of As(III). ArsA is an ATPase that converts membrane potential to ATP, which is usually hydrolyzed by ArsB, making As(III) efflux more efficient. ArsB is an integral membrane protein that pumps As(III) out of the cell (1), however a canonical *arsB* gene is not encoded in the genome of this bacterium. Interestingly, *Nitrincola* sp. has a gene that encodes for the Acr3 extrusion pump (14). Thus, *Nitrincola* sp. strain A-D6 possesses a complete set of genes for dissimilatory oxidation and a system for reducing and extruding arsenic.

### Nucleotide sequence accession numbers.

This whole-genome shotgun project has been deposited at DDBJ/EMBL/GenBank under the accession number JRLB00000000. The version described in this paper is version JRLB01000000.

## References

[1] Slyemi D Bonnefoy V 2012 How prokaryotes deal with arsenic. Environ. Microbiol. Rep. 4:571–586. 10.1111/j.1758-2229.2011.00300.x. 23760928

[2] Cohen SM Arnold LL Eldan M Lewis AS Beck BD 2006 Methylated arsenicals: the implications of metabolism and carcinogenicity studies in rodents to human risk assessment. Crit. Rev. Toxicol. 36:99–133. 10.1080/10408440500534230. 16736939

[3] Páez-Espino D Tamames J De Lorenzo V Cánovas D 2009 Microbial responses to environmental arsenic. BioMetals 22:117–130. 10.1007/s10534-008-9195-y. 19130261

[4] Tsai SL Singh S Chen W 2009 Arsenic metabolism by microbes in nature and the impact on arsenic remediation. Curr. Opin. Biotechnol. 20:659–667. 10.1016/j.copbio.2009.09.013. 19880307

[5] Mukhopadhyay R Rosen BP Phung LT Silver S 2002 Microbial arsenic: from geocycles to genes and enzymes. FEMS Microbiol. Rev. 26:311–325. 10.1111/j.1574-6976.2002.tb00617.x. 12165430

[6] Dimitriu PA Shukla SK Conradt J Márquez MC Ventosa A Maglia A Peyton BM Pinkart HC Mormile MR 2005 *Nitrincola lacisaponensis* gen. nov., sp. nov., a novel alkaliphilic bacterium isolated from an alkaline, saline lake. Int. J. Syst. Evol. Microbiol. 55:2273–2278. 10.1099/ijs.0.63647-0. 16280482

[7] Lohse M Bolger AM Nagel A Fernie AR Lunn JE Stitt M Usadel B 2012 RobiNA: a user-friendly, integrated software solution for RNA-Seq-based transcriptomics. Nucleic Acids Res. 40:W622–W627. 10.1093/nar/gks540. 22684630 PMC3394330

[8] Chevreux B Wetter T Suhai S 1999 Genome sequence assembly using trace signals and additional sequence information, p 45–56 *In* Proceedings of the German conference on bioinformatics (GCB) 99, Hannover, Germany.

[9] Huang X Madan A 1999 CAP3: a DNA sequence assembly program. Genome Res. 9:868–877. 10.1101/gr.9.9.868. 10508846 PMC310812

[10] Aziz RK Bartels D Best AA DeJongh M Disz T Edwards RA Formsma K Gerdes S Glass EM Kubal M Meyer F Olsen GJ Olson R Osterman AL Overbeek RA McNeil LK Paarmann D Paczian T Parrello B Pusch GD Reich C Stevens R Vassieva O Vonstein V Wilke A Zagnitko O 2008 The RAST server: Rapid Annotations using Subsystems Technology. BMC Genomics 9:75. 10.1186/1471-2164-9-75. 18261238 PMC2265698

[11] Lowe TM Eddy SR 1997 tRNAscan-SE: a program for improved detection of transfer RNA genes in genomic sequence. Nucleic Acids Res. 25:955–964. 10.1093/nar/25.5.0955. 9023104 PMC146525

[12] Lagesen K Hallin P Rødland EA Staerfeldt HH Rognes T Ussery DW 2007 RNAmmer: consistent and rapid annotation of ribosomal RNA genes. Nucleic Acids Res. 35:3100–3108. 10.1093/nar/gkm160. 17452365 PMC1888812

[13] Anderson GL Williams J Hille R 1992 The purification and characterization of arsenite oxidase from *Alcaligenes faecalis*, a molybdenum-containing hydroxylase. J. Biol. Chem. 267:23674–23682. 1331097

[14] Mansour NM Sawhney M Tamang DG Vogl C Saier MH Jr 2007 The bile/arsenite/riboflavin transporter (BART) superfamily. FEBS J. 274:612–629. 17288550 10.1111/j.1742-4658.2006.05627.x

